# Mr. Owen in Answer to Mr. Blair

**Published:** 1799-12

**Authors:** William Owen

**Affiliations:** Penton-street, Pentonville


### *To the Editors of the Medical and Phyſical Journal.*

GENTLEMEN,

WITH reſpect to the Welch Hiſtorical Fragment upon the Syphilis, inſerted in your Publication for October laſt, I had no idea that any remarks would have been made, except ſuch as ſhould reſult from the internal evidence of it; but as Mr. BLAIR has been induced to queſtion the correctneſs of that piece, it is incumbent upon me to declare, from the plain ſtile of the original, that the tranſlation conveys the meaning of it verbally throughout; only that, for the ſake of giving a more decent title to the article, as it was thought, the *Brèç Vawr* has been rendered the *great puſtulous eruption*, inſtead of the *great-pox*, which is the literal and popular import of the name.

I remain,

GENTLEMEN,

Your humble ſervant,

WILLIAM OWEN.

*Penton-ſtreet, Pentonville,*

*Nov.* 9, 1799.

